# Plant cell polarity: The many facets of sidedness

**DOI:** 10.1093/plphys/kiad436

**Published:** 2023-08-11

**Authors:** Juan Dong, Jaimie Van Norman, Viktor Žárský, Yan Zhang

**Affiliations:** Waksman Institute of Microbiology, Rutgers, The State University of New Jersey, Piscataway, NJ 08854, USA; Department of Plant Biology, Rutgers, The State University of New Jersey, New Brunswick, NJ 08891, USA; Department of Botany and Plant Sciences, University of California, Riverside, Riverside, CA 92521, USA; Center for Plant Cell Biology, Institute of Integrative Genome Biology, University of California, Riverside, Riverside, CA 92521, USA; Department of Experimental Plant Biology, Faculty of Science, Charles University, 128 44, Prague 2, Czech Republic; Institute of Experimental Botany, Academy of Sciences of the Czech Republic, 165 02 Prague 6, Czech Republic; Department of Plant Biology and Ecology, College of Life Sciences, Nankai University, Tian’jin 300071, China

Plant cell polarity plays a pivotal role in the fundamental processes that dictate plant growth, development, and adaptation. By establishing distinct regions within cells, plant cell polarity is crucial for regulating asymmetric cell divisions, guiding the direction of cell expansion, and determining the spatial distribution of essential cellular components. At the same time, the growth and developmental processes of multicellular organisms create new constraints imposing regulatory feedback on cell and tissue polarity. This intricate organization is essential for various developmental events, such as organ formation, tissue patterning, and vascular network establishment. Additionally, dynamic plant cell polarity is instrumental in responding to environmental cues, enabling plants to adapt to diverse conditions, including light direction, gravity, mechanical stresses, and pathogens attack. The maintenance and reorganization of proper cell polarity are critical for overall plant architecture, as well as for efficient nutrient transport and hormonal signaling. Understanding the mechanisms and regulatory pathways involved in plant cell polarity holds immense promise for enhancing crop productivity, optimizing plant responses to changing environments, and ultimately contributing to sustainable agriculture and ecological resilience. Plant cell biologists embrace captivating challenges, ranging from unraveling the fundamental functions of cells to unraveling the intricacies of plant development and adaptation.

In this Focus Issue, we present 10 *Update* articles that review our current understanding of the many facets of plant cell polarity at the subcellular, cellular, tissue, and organismal levels. Their contents are highlighted below.

## Cell polarity: origination from lateral plasma membrane compartmentation

Virtually, all proteins and protein complexes at the plasma membrane are unevenly distributed, and many form nano- or microdomains to regulate cell signaling. The classic “fluid mosaic model” and “lipid raft model” (Singer and Nicolson 1972; Simons and Ikonen 1997), therefore, have been refined to the dynamic “microdomain” hypothesis in plant cells (Tapken and Murphy 2015; Jaillais and Ott 2019) that emphasize the high compartmentation of the plasma membrane organized through lipid assembly and protein segregation. Increasingly more evidence supports the significant contribution of membrane lipids to the polarization of proteins and protein feedback on lipid nanopatterning in cell morphogenesis. In this Focus Issue, Li et al. from Jingxing Lin's lab summarize the importance of lipids in plasma membrane lateral segregation and provide examples of lipids demonstrating polar distribution in membrane microdomains (Li et al. 2023a). Although lipids alone may cluster in distinct microdomains, it is important to note that the associated proteins, such as the glycosylphosphatidylinositol-anchored proteins, remorins or flotillins, are essential for organizing distinct microdomains. With recent advances in microscopy techniques in detecting finer and faster movements of single molecules at the membrane, it becomes clearer that membrane microdomains act as structural platforms and signaling hubs for the development of membrane polarity (Li et al. 2023b). The underlying molecular basis of the lipid–protein feedback is not fully understood, but protein oligomerization, protein–lipid/calcium ion interactions, and clustering along with specific locally recruited lipid kinase or phosphatase activity likely play important roles in remodeling microdomain properties.

A closely relevant case of membrane compartmentation in establishing cell polarity in possibly all eukaryotes including plants appears to be the small GTPases of the Rho-related GTPases from plants (ROP) family (Smokvarska et al. 2021), a critical regulator of cell polarity in a variety of developmental and growth processes, especially via cytoskeletal and endomembrane effectors (Yang 2008; Ou and Yi 2022; Li et al. 2023a). Here, Pan et al. review recent advances on plasma membrane nanodomains interplaying with ROP signaling often resulting in positive feedback loops to achieve robust polarity in cell morphogenesis and growth (Pan et al. 2023). They first summarize that lipid and protein nanoclustering emerges as a common property crucial for Rho GTPase-mediated signaling in cell polarity across eukaryotes. In plants, the identification/observation of ROP nanoclustering was achieved by super-resolution imaging coupled with single particle tracking of ROP6 in *Arabidopsis* epidermal cells (Platre et al. 2019; Pan et al. 2020; Smokvarska et al. 2020). Pan et al. further elaborate on how various positive ROP-based self-organizing feedback mechanisms contribute to forming spatially restricted signaling nanodomains during cell polarization (Pan et al. 2023). Additionally, membrane biophysical properties defined by the lipid composition provide another sorting platform for ROP nanoclustering and signaling specificity. Importantly, recent studies also show receptor-like kinase (RLK)-mediated regulation, such as the transmembrane kinases (TMKs) and FERONIA, contributes to the organization of nanoclustering and activation of ROP GTPases (Pan et al. 2020; Smokvarska et al. 2023), as discussed by Pan et al. (2023).

While finding the crucial contribution of microdomain to polarizing the plasma membrane in plants is exciting, both Li et al. (Li et al. 2023b) and Pan et al. (Pan et al. 2023) raise outstanding unresolved questions related to understanding the spatiotemporal and dynamic organization of microdomains. Elucidation of these events not only requires high-resolution imaging coupled with single-molecule methodology and quantification, as well as advanced molecular dynamics simulations but also computational methods that facilitate extracting and accelerating image analysis.

## Polarization for function: tip growth, communication, and cell division

Cell and tissue polarity provides an intrinsic mechanism for morphogenetic processes in plants. Polarized plasma membrane domains integrate hormonal, mechanical, and position cues to organize subcellular structures and functions for cell wall remodeling, growth, or defense. As reviewed by Müller (Müller 2023), both tip-growing cells, such as the pollen tube and root hair, and multipolar expanding cells, such as pavement cells, provide excellent platforms to study how regulators of cell polarity orchestrate cellular events in cytoskeletal organization, endomembrane trafficking dynamics, and mechanical signaling to polarize a plant cell for its functional form. Müller first reviews recent advances in understanding tip architecture and ROP-dependent tip growth, highlighting how upstream ROP regulator RopGEFs are regulated by diverse kinases to initiate ROP signaling. She shares insights linking cell wall mechanics, mechanosensing, ROP signaling, and cell wall reinforcing feedback mechanisms in pavement cell morphogenesis that requires the existence of two opposing pathways in the lobe and indentation regions of an expanding pavement cell. Pan et al. (Pan et al. 2023) and Müller (Müller 2023) both highlight new advances suggesting the involvement of auxin-triggered TMK activation of ROP6 nanoclustering on the indentation/concave side (Pan et al. 2020) and brassinosteroids-mediated inhibition of the BR-INSENSITIVE 2 kinase signaling to activate ROP2 on the lobe/convex side (Lauster et al. 2022; Zhang et al. 2022).

Cell polarity signaling orchestrated by ROP GTPases directly or indirectly regulates several key cellular events involved in polar cell growth, including cytoskeletal organization, vesicle trafficking, membrane dynamics, and cell wall remodeling. The rapid tip growth of a pollen tube demands a dynamic actin cytoskeleton that controls cytoplasmic streaming, vectorial vesicle trafficking, and dynamic wall modification. Zhang et al. from Shanjin Huang's group write an Update summarizing recent progress in understanding the regulation and function of the actin cytoskeleton in pollen tube tip growth (Zhang et al. 2023). The current model is based on the observations that at the pollen tube tip, the actin cytoskeleton is divided into (i) shank-localized bundles ending with the cortical F-actin fringe and (ii) apex-localized with highly dynamic very short filaments (Cárdenas et al. 2008), the latter of which is generated from the plasma membrane and recycling endomembranes and arrayed into a unique structure for tip-directed vesicle accumulation (Zhang et al. 2023). The authors also share new insights about how the formin/profilin module promotes actin nucleation and elongation from the apical membrane. In addition, pollen tubes have a tip-focused Ca2+ gradient that is important for polarized growth. They summarize how this gradient is formed and how villins, a group of versatile actin regulatory proteins, coordinate with the Ca^2+^ gradient to control the dynamic and spatial organization of the F-actin network in pollen tubes (Zhang et al. 2023).

In recent years, significant advances have been made in understanding the communication between a pollen tube and the pistil. In this Focus Issue, Ogawa and Kessler discuss a conserved signaling module consisting of the rapid alkalinization factor (RALF) peptide ligands binding to the CrRLK1L/LLG/MLO receptor complex, which is used by the female tissue to manipulate pollen germination and pollen tube growth in *Arabidopsis* reproduction. The emerging theme appears that, at different stages of plant reproduction, these RLKs respond to the RALF ligands to induce autocrine or paracrine signaling that triggers signaling cascades to regulate pollen tube growth orientation and/or integrity (Ogawa and Kessler 2023). Besides pollen tubes, this review article also highlights the polarization of the synergid cells revealed by the enriched distribution of the transmembrane protein NORTIA, an MLO member, to the filiform apparatus, where it acts as a new Ca2+ channel to regulate calcium influx in the synergid cells for pollen tube reception (Ogawa and Kessler 2023). One major challenge toward understanding the RALF/CrRLK1L/LLG/MLO signaling module, according to (Ogawa and Kessler 2023), resides in its cooption for a wide range of signaling processes—how functional specificity can be achieved in root development, plant–pathogen interactions, and responses to stresses and hormones by the components of this rather general signaling module.

Cell polarity provides one major mechanism to specify geometrical asymmetry and division orientation in plant cell formative divisions (Guo and Dong 2022; Hartman and Muroyama 2023). To divide in a developmentally meaningful manner, multiple levels of polarity in tissues and cells contribute to a final oriented cell division (Glanc 2022). In maize stomatal development, ROP proteins are polarized with the receptor proteins to specify the asymmetric division of the subsidiary cells (Humphries et al. 2011). Müller updates the recent progress in the analysis of ROP signaling for formative cell divisions in early phloem progenitor and root meristem cells (Müller 2023). Several components and regulators of the ROP signaling pathway show dynamic and specific relationships with the assembly and disassembly of the preprophase band in the cortical division zone (Stöckle et al. 2016; Kumari et al. 2021). Inspired by the recent work demonstrating ROP's function in spatial patterning of cell division in the liverwort *Marchantia polymorph* (Rong et al. 2022), she suggests ROP GTPases could contribute to division orientation more broadly (Müller 2023), possibly linking local cell behavior to tissue wide polarity field (Mansfield et al. 2018).

In plants, cytokinesis, the final stage of the cell cycle, significantly differs from animal cells due to the presence of a rigid cell wall surrounding the protoplast. Vesicles containing cell wall materials, such as pectins or xyloglucan polysaccharides, callose, and cellulose synthases, are transported to the equatorial region of the cell by the phragmoplast vesicles. Callose is a transient constituent of the growing cell plate and provides a conserved mechanical stabilization role. Importantly, the formation of callose in cytokinesis requires polar synthesis at the future plasma membrane at the expanding cell plate by the callose synthase (CalS). Ušák et al. provide a comprehensive evolutionary insight into the roles of callose in cell wall remodeling and present the conserved phylogeny of CalS proteins across the plant kingdom (Ušák et al. 2023). CalSs are a family of large, integral membrane proteins (200-kD), whose delivery involves activities of the endomembrane system. Although the molecular mechanisms underlying the polar delivery of CalS and callose deposition are not clear, the authors summarize the spatiotemporal delivery of plant cellulose synthase (CESA) and compare it to that of fungal chitin synthase, both of which as CalS are large integral proteins mediating polysaccharide synthesis in the cell wall (Ušák et al. 2023). Ušák et al. also raise several significant questions to be addressed in the future. For example, what are the signaling components contributing to the spatiotemporal localization of the CalS proteins? Thus, more insights into the regulation of callose synthesis, enzymatic activity, directional delivery, and functional specificity at the molecular level are much needed.

## From 3-d to 4-d and from local to global: cell polarity in plant development

Each individual cell contains a variety of organelles, which can be membrane-bound or membraneless, and each organelle performs a set of specific functions as hubs of metabolisms and elements of a structure. Most often, we use 3 spatial dimensions to describe the morphology and localization of an organelle at a given time. In this Focus Issue, Hickey et al. discuss the 4th dimension—time, and how changes in organelle structure and activity in responding to external cues generate a 4th dimension of plant gradient/polarity in development. This review highlights the significance of organellomic gradients in the time dimension (changes in organelle abundance, morphology, and functions) and the context of developmental or stress responses. They also identify quantifiable parameters that can be used for assessing changes in the organelles, such as auto-phagosomes, cytoskeleton, endomembrane compartments, lipid bodies, mitochondria, peroxisomes, etc (Hickey et al. 2023). These changes can be measured in the organelle's biogenesis, diversity, and abundance during developmental differentiation or stress situations.

One fundamental feature determining the formation of a functional multicellular body in plants is the establishment and development of body axes that coordinate with cell and field polarities (Ramalho et al. 2021). The apical-basal axis is initiated in early embryo development, and cells in the shoot apical meristem (SAM) divide and differentiate to give rise to most of the aboveground shoot tissues. Wang and Jiao revisit the classic CLAVATA-WUSCHEL (CLV-WUS) signaling module and update new advances in identifying additional regulators, including the receptor-like cytoplasmic PBL (PBS-1 LIKE) kinases and the HAIRY MERISTEM GRAS transcription factors, for their roles in maintaining SAM homeostasis (Wang and Jiao 2023). The paper further discusses the roles of auxin signaling and polar transport in the shoot apex, highlighting the highly dynamic changes in the localization of transporters, auxin levels, and auxin signaling during the shoot organogenesis (Wang and Jiao 2023). One obvious direction for future inquiry is suggested to explore the coupling mechanism between the highly dynamic auxin distribution and signaling with the CLV-WUS feedback loop. In addition, with the recent advances in single-cell transcriptome analysis in plants, developmental trajectories of several cell types in the *Arabidopsis* shoot apex have been constructed, and high cellular heterogeneity of SAM appears to be a common feature in both monocots and dicots (Wang and Jiao 2023). Due to the low abundance of SAM cells, the power of single-cell analysis can be improved by enriching the cell type and possibly by combining with fluorescence-activated cell sorting (Otero et al. 2022) and single-nucleus RNA-seq (Long et al. 2021).

The overall apical-basal axis of the plant body extending from the tip of the shoot toward the root aligns well with the direction of leaf vein formation. This system provides a unique opportunity to reveal the molecular mechanism underlying the coordination between cell polarity and body axis in plant cells that are restricted from migration and movement in development. While polar transport of the plant hormone auxin has been long thought to be required for vein formation, Scarpella discusses that the combination of polar transport and axial diffusion of auxin can account for leaf vein formation and other processes in plant development (Scarpella 2023). Scarpella first presents the “canalization hypothesis” formulated 50 years ago by Sachs who proposes auxin transport induces a positive feedback response so that the movement of auxin through a file of cells increases its capability to transport auxin (Sachs 1981). He then presents accumulating experimental evidence and simulation results supporting that, besides PIN-mediated polar auxin transport, auxin diffusion through plasmodesmata intercellular channels is required for vein formation and their proper connectivity. The new model suggests that auxin initially diffuses all over a young leaf through plasmodesmata, and this facilitated diffusion mechanism becomes gradually limited to the axis of the developing veins. Thus, the pattern formation of vascular strands requires auxin signaling, polar auxin transport, and facilitated auxin diffusion through the plasmodesmata (Scarpella 2023). A priority question to be addressed in future research is probably the molecular basis of auxin-controlled plasmodesma permeability.

Finally, cell polarity in plants is manifested by the polarization of proteins that impact anisotropic cell growth and various plant growth processes. Systems biology and computer models progress along with accumulating scientific discoveries about how genetic, chemical, and mechanical inputs determine cell polarity and regulate polarity-dependent processes. Marconi and Wabnik provide a review summarizing several computer models of cell polarity-related processes in plants, including auxin-mediated cell polarity, mechanical feedback on tissue polarity, and cell polarity for cell division (Marconi and Wabnik 2023). As auxin polar transport in plants largely relies on the polarization and activation of PIN efflux transporters, auxin has been identified as the predominant regulator of cell, tissue, and overall polarity in plants—a plant morphogen. Considering the self-enforcing feedback between auxin and PINs, the 2 models of auxin-induced PIN polarity (“flux-based model” and “concentration-based model”) define PIN polarity as a nonlinear function of auxin levels, but neither alone can fully simulate all the experimental observations. The authors further provide updates on recent models attempting to reconcile the gaps by implementing new parameters, such as the direction-sensing of the cell or additional regulatory and polarizer proteins in the cell. Besides the models focusing on auxin chemical input in generating polarity, there are models simulating how cell polarity arises from external mechanical forces—mechanical coupling mediated by a turgescent cell wall network. In the mechanical feedback model, high local auxin concentration relaxes the elasticity of its own cell wall but not that of the neighboring cells, resulting in increased mechanical stresses that influence microtubule orientation (i.e. also cellulose microfibril deposition), PIN polarization and morphogenesis (Heisler et al. 2010). Finally, Marconi and Wabnik summarize new advances in developing models to simulate mechanical feedback on tissue-level polarity fields to generate a wide range of shapes in 3D. Indeed, computation models allow scientists to investigate new implications, point to the yet hidden components, and test new hypotheses and predictions (Marconi and Wabnik 2023). With the help of additional experimental data and new computer techniques, researchers will have more powerful tools to develop comprehensive and possibly even predictive models integrating genetic, biochemical, and mechanical information to investigate cell polarity and its consequences.

## Closing remarks

With heartfelt gratitude, we acknowledge the collaborative endeavors of our editorial team throughout the past 12 months, which have led to the creation of this remarkable Focus Issue. We extend our sincerest appreciation to all the authors and reviewers for their invaluable contributions. Although not exhaustive in its coverage of the field of plant cell polarity, the *Updates* and *Research Articles* presented here have illuminated diverse facets of this crucial area of research and underscored the substantial strides being taken in understanding plant cell polarity mechanisms. In conclusion, our earnest desire is for you to relish reading these papers as much as we have cherished assembling them.

## Dive Curated Terms

The following phenotypic, genotypic, and functional terms are of significance to the work described in this paper:

ROP2 Gramene: AT1G20090
ROP2 Araport: AT1G20090

plant cell AmiGo: PO:0009002

Feronia Gramene: AT3G51550
Feronia Araport: AT3G51550

MERISTEM AmiGo: PO:0009013

TMK Gramene: AT5G59440
TMK Araport: AT5G59440

ROP6 Gramene: AT4G35020
ROP6 Araport: AT4G35020
